# Towards a Planetary Health Impact Assessment Framework: Exploring Expert Knowledge and Artificial Intelligence for a RF‐EMF Exposure Case‐Study

**DOI:** 10.1002/bem.70038

**Published:** 2025-12-19

**Authors:** Magdalini Stefanopoulou, Tabea S. Sonnenschein, Florence Poulletier de Gannes, Simon Scheider, Roel Vermeulen, Martin Röösli, Anke Huss

**Affiliations:** ^1^ Institute of Risk Assessment Sciences Utrecht University Utrecht the Netherlands; ^2^ Human Geography and Spatial Planning, Faculty of Geosciences Utrecht University Utrecht the Netherlands; ^3^ Julius Centre for Health Sciences and Primary Care, University Medical Centre Utrecht Utrecht University Utrecht the Netherlands; ^4^ Laboratoire de l'Intégration du Matériau au Système (IMS) Université Bordeaux Talence France; ^5^ Department of Epidemiology and Public Health Swiss Tropical and Public Health Institute Basel Switzerland

**Keywords:** artificial intelligence, expert elicitation, knowledge graphs, mobile telecommunication technologies, planetary health

## Abstract

While recent WHO systematic reviews have comprehensively assessed the direct health effects of radiofrequency electromagnetic field (RF‐EMF) exposure, its potential indirect impacts on human health via ecosystem disruption remain unstudied. Therefore, we propose a Planetary Health Impact Assessment (PHIA) approach, which incorporates both direct and ecologically mediated pathways. Developing the underlying framework requires a method for organizing and visualizing complex, interdisciplinary knowledge. This study explores an approach for constructing a PHIA framework in the form of knowledge graphs (KGs). Using RF‐EMF exposure from mobile telecommunication technologies as a case study, we developed an expert‐based KG in collaboration with 12 specialists. We further evaluated the potential of an artificial intelligence (AI)‐based tool, incorporating Natural Language Processing (NLP) and Deep Learning, to extract relevant information from scientific literature and generate KGs to explore ways to enhance the expert‐based approach. Experts developed and visualized jointly the hypothesized pathways linking RF‐EMF exposure to direct health effects on organisms and indirect effects on human health through ecological consequences. The AI tool quickly processed large volumes of literature and visualized it into KGs with varied structures but required extensive expert validation due to limitations in precision and context sensitivity. The expert‐based KG can serve as organizer of the available knowledge and as a first step in PHIA development. While AI tools offer potential for exploratory analysis, they currently require substantial human oversight and cannot replace expert judgment. The resulting KGs also identified possible gaps in the scientific literature.

## Summary

- RF‐EMF exposure from mobile telecommunications was used as a case study to explore which direct and indirect pathways might affect human health.
- An expert‐based knowledge graph and an AI‐based tool were used to organize and visualize complex, interdisciplinary knowledge on RF‐EMF exposure effects.
- The expert‐based approach provided greater contextual accuracy than the AI tool and helped identify literature gaps, supporting the development of a Planetary Health Impact Assessment (PHIA) framework.

## 1 Introduction

The rapid pace of human impact on Earth's natural systems contributes to the global burden of disease (Myers 2017; Whitmee et al. 2015). This, combined with the realization that indirect, or longer‐term, effects of ecosystem disruption may have significant impacts on human health (Young and Hsiang 2024), has changed the focus towards a new research field: planetary health. The concept of planetary health recognises that human health and civilisation are dependent on flourishing natural systems and their wise stewardship. Planetary health seeks to address the public health impacts of human‐driven environmental changes through interdisciplinary approaches (Whitmee et al. 2015).

During the past decade, exposure to radiofrequency electromagnetic fields (RF‐EMF) from mobile telecommunication technology has become a near‐ubiquitous anthropogenic factor in the environment. Although the introduction of new technologies in the past, such as the transition from 3 to 4 G, led to changes in exposure patterns (Urbinello et al. 2014), current evidence indicates that during the past several years, exposure patterns have not substantially changed (Loizeau et al. 2023; Beláčková et al. 2025). However, the recently introduced fifth generation (5 G New Radio [NR]) operates in the higher frequency parts of the electromagnetic spectrum, resulting in higher absorption of the exposure at the body's surface for humans.

For smaller animals like insects, 5 G deployment may lead to higher absorption rates due to resonance effects, with unclear consequences (Thielens et al. 2018). Given the vital role insects play in maintaining ecosystem health, any effects of RF‐EMF exposure could also indirectly affect human health. Recent efforts by the WHO have produced a series of systematic reviews to evaluate the available evidence on health effects of exposure to RF‐EMF, focusing on direct effects on human health. However, established methods cannot address the potential for indirect health effects, such as those mediated through impacts on non‐human organisms or ecological systems. Addressing this gap requires a broader, integrative framework that incorporates both direct and ecologically mediated pathways between environmental change and human health.

Building on this foundation, we believe the next logical step is to conduct a broader Health Impact Assessment (HIA). Planetary Health Impact Assessment (PHIA) has been proposed as a novel and more inclusive approach (Osofsky and Pongsiri 2018) to guide decision‐making and, subsequently, sustainable policies. While promising, PHIA presents methodological challenges due to the complex and interconnected nature of human and environmental systems. Developing a transparent, reproducible, and actionable methodology is essential to move this concept forward. Due to its potential environmental implications, RF‐EMF is a suitable case study to develop a PHIA.

Following the traditional HIA, the first step towards a PHIA should begin with synthesizing available evidence into a framework (Lock 2000), for instance a knowledge graph (KG), a representation of a network of real‐world entities and their relationships (Tamašauskaitė and Groth 2023). This organising principle could be a useful tool since it allows summarizing and visualising information related to investigated direct and indirect pathways linking human‐driven environmental changes to human health. The resulting KG could serve as a communication tool, aiding the understanding of these complex relationships, the research prioritization by highlighting research gaps, and support PHIA framework's further development by allowing the integration of additional information and support risk assessment.

Nevertheless, organising available knowledge into a KG is a complex task. Experts' knowledge elicitation could be a starting point for constructing such an overview since experts can provide updated insights and interpret complex relationships. However, their availability may be limited, and their perspectives shaped by and restricted to their areas of expertise. Unlike most existing review tools, which primarily support article screening and tagging, AI tools that extract knowledge from published literature may be able to address these challenges. These AI tools can efficiently process large volumes of literature and are not influenced by biases towards particular domains. However, currently available AI tools may suffer from restricted insights of complex relationships, or from being unable to take study quality into account for an overall assessment of the scientific evidence.

In the subsequent sections, we describe an approach for developing this KG through expert elicitation. Additionally, we explore the ability of an AI tool to extract the aforementioned information from scientific literature and structure it in KGs, in an attempt to explore its potential to enhance or replace the expert‐driven approach. For this analysis, we used an automated tool trained to extract evidence from scientific literature which utilizes Natural Language Processing (NLP) and Deep Learning (Sonnenschein et al. 2024). Given that the current data on the effects of RF‐EMF on organisms is inconclusive and/or limited with no studies directly linking these effects to potential indirect impacts on human health through ecological consequences, we aimed to retrieve both hypothesized and reported associations. This study is guided by two key research questions:

1. Can we use experts' knowledge to create overviews of complex possible direct and indirect effects on human health using RE‐EMF exposure as case‐study?
2. How does the AI tool perform when tasked to construct similar overviews using scientific literature, and what insights can this provide?

## 2 Methods

### 2.1 Experts‐Based KG

Our primary approach required experts' knowledge elicitation and judgment regarding the nodes and edges of the KG. The recruitment of the experts and the knowledge elicitation in form of interviews took place between December 2023 and May 2024. This process was divided into three phases: (1) defining our questions; (2) selecting and recruiting the experts; and (3) conducting the actual elicitation. We consulted the principles and recommendations on Expert Knowledge Elicitation on Food and Feed Safety Risk Assessment published by EFSA (European Food Safety Authority [EFSA] 2014). The first phase of the process involved defining the key questions for the experts' workshops. To do so, we first reviewed the published literature to assess what is currently known and to help formulate the key questions for the experts, which are presented in Table ST1. Based on the published literature, we additionally developed an initial version of the KG, which served to guide and structure the discussions during the workshops (Figure SF1). Then, we continued by identifying suitable experts using a combined network and literature‐based approach: First, we invited scientists with relevant expertise in the remit of ETAIN. Second, we identified additional experts beyond the project network by searching the author lists of key publications, such as WHO systematic reviews. Invited experts were asked to suggest colleagues who might be able to fill in information deficits in our KG in cases we had difficulty locating areas in specific fields (e.g., plant ecology) (European Food Safety Authority [EFSA] 2014). We invited experts from a broad range of research areas, including epidemiology, agriculture, biology, entomology, physics, and so forth, from both the academia and outside academia, regardless of geographic location or spoken language. In total, we contacted 16 experts from various fields and 12 of them responded positively to our invitation. An overview of participating experts, including their research areas is provided in Table ST2. Both online and face‐to‐face workshops, with participation of single or multiple experts, were organised. Each workshop was focused on just one part of the KG, and experts from the same field participated in the same workshop, in order to efficiently cover a wide range of insights. Participating experts always received the most recent version of the KG in advance. Workshops were supervised by two facilitators to monitor discussion. The final KG represents the aggregated opinion of all the experts. After each workshop, the experts received the KG as it had been formulated during the session and were encouraged to share updates to ensure that any new insights were integrated in the KG.

### 2.2 Al Tool‐Based KGs

Subsequently, we used an AI tool to automatically extract information from scientific literature and structure it in KGs. Briefly, this process included a manual literature search to locate relevant literature, followed by title and abstract screening with ASReview, the automated extraction of direct health effects for various study groups, including humans and different types of organisms, as well as of the potential indirect effects through ecological consequences, the synthesis of the extracted information and, finally, the visualization in KGs.

#### 2.2.1 Literature Search and Curation

We searched PubMed and Web of Science for scientific literature. Two separate search strategies were designed to identify studies investigating (1) the direct effects of RF‐EMF exposure on various organisms and (2) indirect effects on human health. We applied filters to only include reviews and systematic reviews with or without meta‐analysis that included either observational or experimental studies conducted on laboratory or natural settings, published from 2000 onward. Specifically for indirect effects, we designed a search strategy to identify studies reporting effects on human health due to disruption of ecosystem services offered by plants, birds, and insects, as experts agreed that the most ecologically relevant direct effects were linked to them. Only the searches for “population,” “richness,” “composition,” and “diversity” showed relevant publications. Our complete search terms and Boolean operators used for each strategy are provided in Tables ST3 and ST4. Papers identified by the search were then screened and selected using the ASReview tool (ASReview LAB version 1.6.2) based on title and abstract, following specific inclusion criteria, separately for each dataset. We stopped screening after a number of consecutive records were found irrelevant or after approximately 20%–30% of the dataset was screened, whichever happened first. These are common stopping strategies for active learning practices. Table ST5 summarizes the ASReview screening outcomes. Duplicates were removed prior to screening. Figures SF2 and SF3 present the flowcharts for the two separate search strategies. For studies on the direct effects, we included papers focusing on RF‐EMF in the range between 30 MHz and 3 GHz, as well as on the higher frequency range that 5 G will partially operate on in the future (from 3 to 60 GHz). Studies that examined generally electromagnetic fields without specifying the frequency range or that included other frequency ranges (e.g., extremely‐low electromagnetic fields) were excluded in order to facilitate the usage of the automated tool. Eligible studies reported outcomes related with the health or behavior of the studied organisms. Only peer‐reviewed literature was included with the following exception. Specifically following experts recommendations, we added in our resulted papers, the report by Arno Thielens (Thielens 2021) on the environmental impact of 5 G, published by the Scientific Foresight Unit (STOA), as it included updated data regarding the environmental impact of RF‐RMF. For the indirect effects, we included peer‐reviewed studies that solely focused on different aspects of human health and wellbeing, but we excluded studies investigating economic or social impacts on humans. Additionally, we excluded studies focusing on infectious diseases (e.g., Influenza, Malaria, West Nile virus) or on the effects of green spaces.

#### 2.2.2 Automated Information Extraction

We used an automated knowledge extraction tool as described by Sonnenschein et al. (2024). Data extraction was performed in Python (version 3.12.3). This process was carried out twice, for studies evaluating direct effects of RF‐EMF on different organisms, and for indirect effects of RF‐EMF on human health. For each set of studies, manual labeling, tool training, label prediction, extraction, harmonisation, and visualization were done separately to fit the distinct types of information, since currently there are no studies to investigate them simultaneously.

### 2.3 Manual Labeling, Tool Training, and Label Prediction

We started with the manual labeling of a subset of papers based on the type of information we wanted to extract. We focused on information related on the exposure type, direct and indirect health effects, study groups, study types, reported and hypothesised relationships, and so forth. The labeling process was carried out by a researcher following a labeling manual based on the approach and instructions used by Sonnenschein and colleagues (2024). The researcher was advised to label information consistently through the whole text. If the best representative label for a piece of information was unclear, then the researcher was advised to go back to the original research to ensure accurate labeling. Additionally, to ensure that the labeling manual was clear and the labeling was consistent, we tested the inter‐annotator agreement of the manually labeled papers by having two additional researchers to label three papers, which resulted in 92·3% agreement and Cohen's kappa coefficient 0·53. Specifically, for the direct effects, we created six labels based on the type of information we wanted to be extracted from the papers and for the indirect effects, we created five labels. Tables ST6 and ST7 describe the labels developed for the direct effects and indirect effects, respectively.

The labeled data were used to train the BERT‐Name Entity Recognition (NER) model. We used a BERT pretrained cased base model for token classification from Python's transformers package (Wolf et al. 2020) and trained the model. Ten percent of the labeled data was used for validation. We trained and evaluated the predictive performance of the BERT‐NER model using F1‐score. Figures SF4 and SF5 present the learning curves for BERT‐NER model's performance in predicting labels over time related on the direct effects of RF‐EMF exposure on organisms and the possible indirect effects on human health due to potential ecological consequences, respectively. Table ST8 presents the used training parameters and F1‐scores for the best‐performing models.

Following the initial BERT‐NER predictions, we manually reviewed a subset of the predicted instances and used them to train multiple supervised classification models. Repeated stratified cross‐validation was applied to optimize the models and identify the true instances from the predicted set. Among the models we tested, the gradient boosting showed the best performance and was used to predict the final set of unique and complete instances for both direct and indirect effects. The F1‐scores for the gradient boosting are presented in Table ST8. The specific numbers of papers and sentences manually labeled, predicted instances, instances used for training, and final outputs are reported in Table ST9.

### 2.4 Extraction, Harmonisation, and Visualisation

The extracted instances underwent an automated harmonization process, followed by a manual revision and aggregation into broader categories. Specifically for the direct health effects, to ensure that the health outcomes were classified as accurate and consistent as possible, we created broader categories according to the coding of outcomes as has been listed in previous literature (Cucurachi et al. 2013; Karipidis et al. 2023; Pophof et al. 2023) and general recommendations given by an expert. The harmonization reduced the 2265 unique health effects into 90 categories, which were meta‐classified into 37 meta‐categories. For the exposure types, the harmonization reduced the 1271 unique exposure types into seven categories. For the study groups, the initial 20 unique study groups were aggregated into 12 larger categories. For the association type, we followed a different way of classification: Initially, we classified the extracted information into two categories based on whether they were describing a hypothesized or a reported association. Subsequently, the instances that were grouped as “reported association” were categorized based on whether this association was or was not statistically significant, followed a positive or negative association and whether it had been described consistently in the literature or not. For the indirect effects, 81 unique direct effects were classified into five broader categories, 37 unique ecological consequences into 12 broader categories and 142 unique indirect effects into 14 categories. For the indirect effects, we had 23 unique study groups in total that were classified into six larger groups. The relationships between the direct effects and the ecological consequences or indirect effects were classified in a similar way to the classification applied to the direct effects dataset.

Before visualization, output tables were cleaned by removing any irrelevant extracted instances (e.g., related to extremely‐low‐frequency fields, visible light, ionizing radiation, etc.) and the manual correction of words that were not extracted correctly. For direct effects, to facilitate visualization, we selected only instances where the study group had been extracted. Cleaned tables were imported in the software Gephi (version 0.10.1) for visualization.

As a test case, OpenAI's ChatGPT was also tasked to extract and visualize the same information described above. More details related to the process followed and the obtained output can be found in the Supporting material (pp. 2–3, Table ST10, Figure SF17).

## 3 Results

### 3.1 Experts‐Based KG

**Figure 1:**
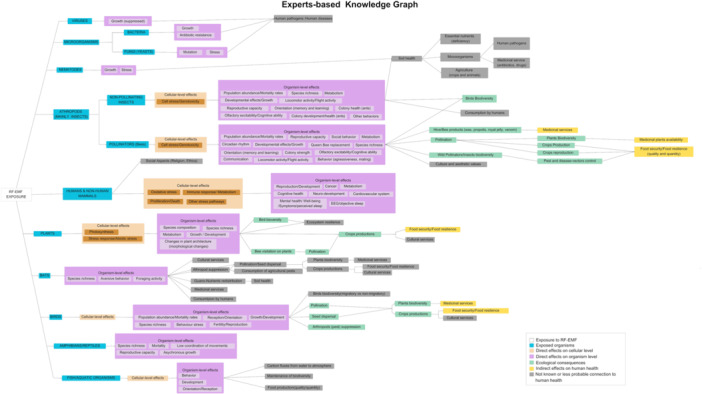
Experts‐based Knowledge Graph. Knowledge graph as constructed by the panel of experts. The experts highlighted some ecological consequences and indirect effects, as well as social, cultural, and aesthetic impacts on human life, in gray to indicate uncertainty about their practical influence on human health.

Figure 1 depicts the final version of the KG constructed by the expert panel. Experts visualized possible direct and indirect pathways leading from exposure to RF‐EMF to human health, for 10 groups of organisms. Humans and non‐human mammals were grouped together. Potential direct health effects linked to each type of organisms were grouped into two levels: cellular‐level effects and organism‐level effects. Direct effects were linked with potential ecological consequences based on the ecosystem services that each type of organism offers, and finally with hypothesized indirect effects on human health. The experts agreed that only direct effects on insects, plants, and birds could lead to ecologically relevant consequences and potential indirect effects on human health. Specifically, direct effects on other organisms, such as nematodes, fish and aquatic organisms, etc. were considered less probable under real‐life conditions, reducing the likelihood of ecosystem disruption and subsequent health impacts. This judgment was based on the observation that for some organisms, although direct effects have been observed in experimental settings, these occurred at much higher exposure levels than those typically found in the environment. Additionally, RF‐EMF would be strongly attenuated in soil or water, making direct effects on certain organisms less likely.

### 3.2 Automated Knowledge Extraction Tool‐Based KGs

**Figure 2:**
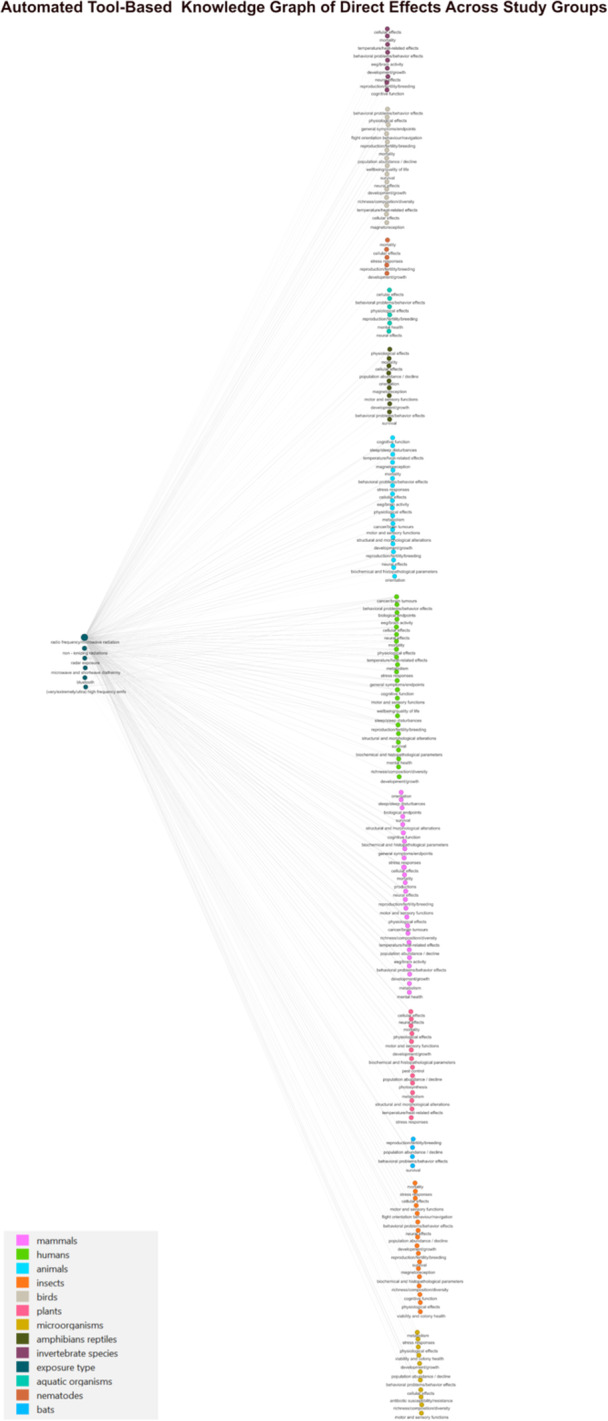
Automated tool‐knowledge graph of direct effects across study groups. Knowledge graph as constructed by the automated tool, depicting the mega‐categories of health effects grouped by study group. The edges connecting the nodes represent the extracted hypothesized and reported associations.

**Figure 3:**
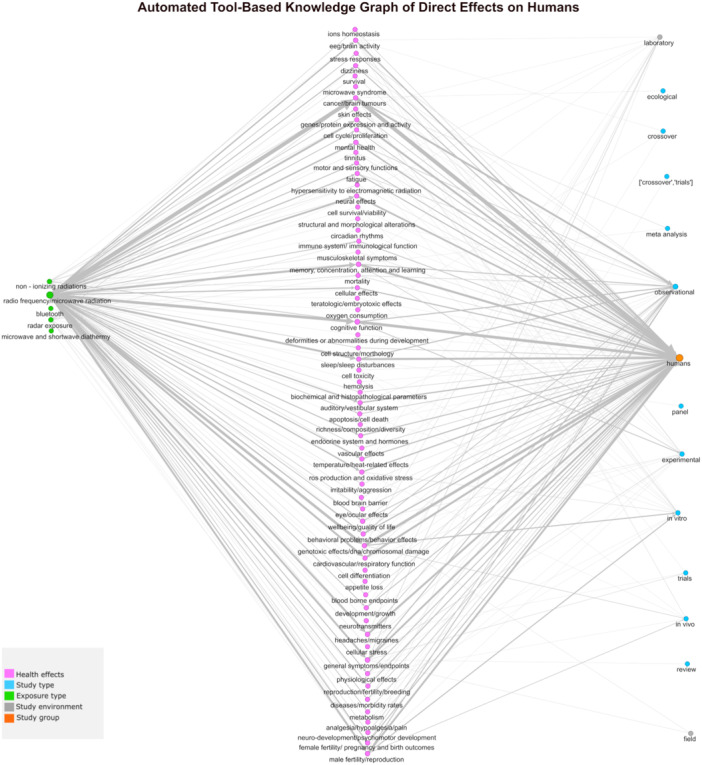
Automated tool‐based knowledge graph of direct effects on humans. Graph restricted to the extracted information related to study group of humans. The edges connecting the nodes represent the extracted hypothesized and reported associations. The edge width represents the number of articles stating the association with minimum 1 and maximum 31 articles.

**Figure 4:**
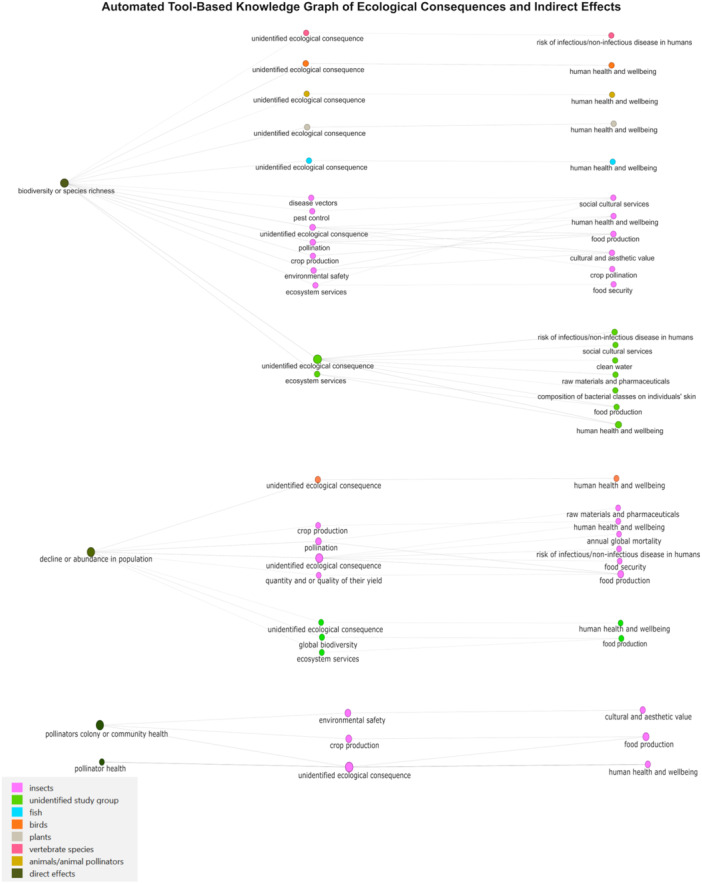
Automated tool‐based knowledge graph of ecological consequences and indirect effects. Knowledge graph as constructed by the automated tool, depicting the ecological consequences and indirect effects on humans. The edges connecting the nodes represent the extracted hypothesized and reported associations. The extracted ecological consequences and indirect effects were colored to correspond on the group of organisms linked to the direct effects. The “unidentified study group” indicates that the study group was either not mentioned in the extracted sentence or not extracted by the tool. The “unidentified ecological consequence” indicates that the ecological consequence was either not mentioned in the extracted sentence, not extracted by the tool, or was unknown.

We identified 97 publications on the potential effects of RF‐EMF on humans and various organisms and 13 reviews on potential ecological consequences and their effects on human health related to insects, birds, and plants. After automated extraction and cleaning, we compiled a table of 4215 unique instances that reported or hypothesized associations between RF‐EMF and direct health effects and 232 unique instances on potential indirect effects (Table ST8). Figures 2 and 4 show the resulting KGs. Figure 2 illustrates a simplified version of the extracted direct health effects. The figure does not show data on study types (e.g., observational or experimental study type), study environments (e.g., field or laboratory study), or potential moderators (e.g., sleeping habits). Individual graphs per study group, showing information on the study types and study environments were also constructed; Figure 3 shows the corresponding graph constructed for humans. Figures SF6–SF16 show corresponding graphs for the remaining study groups. Figure 4 visualizes the extracted information representing hypothesized pathways for direct effects, specifically effects on population, species diversity, pollinators community/colony health, and general pollinators health, linked to ecological consequences due to ecosystem service disruptions. These were subsequently connected to indirect effects on humans. If the study group or ecological consequence linking the direct effects on organisms to the indirect effects on humans was not mentioned in the original text, not extracted by the automated tool, or was unknown, it was added to the KG as a node labeled as “unidentified.”

## 4 Discussion

In this paper, we suggest an approach to develop a first version of PHIA framework in the form of a KG through experts' knowledge elicitation. Our methodology involved experts from various fields. The resulting KG visualizes hypotheses related to direct health effects of RF‐EMF on several organisms and potential indirect impacts on human health through ecological consequences.

The involvement of experts from multiple disciplines helped to capture the interdisciplinary part of our research question: the potential relationships between the direct effects, ecological consequences, and indirect effects on human health. Although our experts‐based KG was not developed using collaborative ontology engineering, our process aligns with some of its core principles, ensuring the reliability of the extracted information (Simperl and Luczak‐Rösch 2013). The resulting KG was the result of a consensus‐driven process between the involved experts. This ensured that conflicting perspectives were discussed, making the resulting KG a balanced representation of current well‐supported hypotheses regarding direct and indirect effects of RF‐EMF exposure. Additionally, workshops were supervised by two facilitators keeping track of the reasoning behind the decisions, ensuring transparency and traceability in how the KG was constructed. Furthermore, experts were encouraged to share their updates, with the intention to integrate emerging insights. While we combined different recruitment approaches to improve inclusivity and reduce bias, some limitations remain. A key limitation of the KG was the small number of participating experts, as the synthesized information depends on the areas of expertise and individual judgments of the experts. This limitation is particularly relevant given that some experts were recruited through a snowballing sampling method, which may have influenced the diversity of perspectives represented. Moreover, despite the efforts to include experts from diverse geographic regions and disciplines, participation was limited to those who were available and willing to engage, which could also have influenced the range of represented perspectives.

Additionally, we used an AI tool to test its ability to automatically extract and visualize the same information from scientific publications, in order to explore ways to enhance or possibly replace the experts‐based approach. The AI‐tool managed to extract and visualize information, however, it had clear strengths and limitations. Its key advantage was its ability to extract and synthesize information from many publications in very short time. The description of the direct effects by Sonnenschein's tool (Sonnenschein et al. 2024) was more detailed, which could mean that it extracted potential direct effects from the literature that might have been missed in our experts‐based KG because we failed to detect the adequate experts. However, Sonnenschein's tool (Sonnenschein et al. 2024) could not extract ecological consequences for several studied organisms. This limitation could be attributed either to our inability to retrieve relevant publications, or the fact that these links were already known to the experts but have not yet been reviewed and published in the scientific literature. Additionally, since the total weighted F1‐scores for our best models indicated moderate performance, it is possible that information may have been missed, or that information we aimed to extract was not well defined. Of note, Sonnenschein's tool was originally designed to extract evidence, not hypotheses. Another relevant limitation was its inability to evaluate the quality of the input studies. Although we included only reviews and/or systematic reviews as knowledge sources, there may still be low‐quality reviews, especially since the introduction of Risk of Bias evaluation is a relatively new technique, which the tool was unable to distinguish. Therefore, the extracted information was as good as the input papers that have been provided by the human observer. When it comes to the resulting KGs, Sonnenschein's tool allowed us to visualize both the aggregated and initially extracted information depending on the level of details desired. However, the resulting KGs structure were not similar to the experts‐based KG. Since there were no studies investigating both direct and indirect effects on humans simultaneously, the output included two separate types of KGs: one for the direct effects and one for the indirect effects. We did not merge the output KGs manually, as we could not rule out subtle mismatches in the definitions of the same nodes across studies (e.g., diversity/species richness), making it unclear whether they should be treated as identical. Another limitation of Sonnenschein's tool (Sonnenschein et al. 2024) is that it came at the expense of an expert necessary to program the tool. However, it allowed us to check the extraction and synthesis of the information, as we could supervise and control the different parts of the algorithm. Regarding the ChatGPT, our test case showed that although the resulting KG included all the intended information, there were certain challenges that made it impossible to validate the reliability of the results. More details are included in the Supporting material (pp. 2–3).

Although current AI tools accomplish a great amount of work that would otherwise require considerable time and effort, they still need further development to operate independently. Considering that, we believe that for fast‐developing fields and interdisciplinary topics that are not yet extensively researched, where published evidence remains limited, AI‐tools may serve as exploratory tools. They can provide a broad synthesis of available knowledge, but since this information is often unfiltered, experts should discern the most relevant insights and refine the output into actionable knowledge. Future advancements should focus on improving the quality and applicability of these tools. Additionally, in this study, we used the ASReview tool, which uses active learning to facilitate the literature research by prioritising the most relevant studies. Although ASReview significantly reduces screening time, human input is still essential for training the model and making the final selection. Therefore, a reliable AI tool that can independently access scientific literature databases and conduct a comprehensive literature search is needed. Our experience managing a large body of literature highlights the urgent need for such tools,not only to automate literature retrieval but also to evaluate the quality of the screened papers. As it has been described previously (Sonnenschein et al. 2024), training the tools to extract knowledge related to the methodology and evaluate studies based on methodological criteria could solve the quality challenge and it would also allow to use original studies as input. Additionally, we followed Sonnenschein et al. (2024) approach and we used a general‐domain BERT model rather than a domain‐specific model such as the PubMedBERT (Gu et al. 2020), as we used a broad range of literature beyond the biomedical field, such as environmental sciences and ecology. Therefore, a general model was more suitable to extract information related to various organisms and study types. However, in the future, the usage of a domain‐specific model such as PubMedBERT (Gu et al. 2020) could potentially improve the extraction accuracy in literature datasets that are mainly biomedical or epidemiological.

## 5 Conclusions and Future Development of PHIA Framework

By constructing a KG through experts, we suggest an approach to summarize and synthesize knowledge from multiple disciplines, enabling a clearer understanding of the interconnected pathways through which RF‐EMF exposure may impact ecosystems and human health. In this context, current AI‐tools could serve as exploratory tools that can guide expert discussions and workshops rather than replace them.

As research proceeds, any KG will continue to change as additional components are incorporated, and less relevant components eliminated in an attempt for simplification and ultimately prioritization of health impacts. Therefore, the next step in developing the PHIA framework should involve transitioning from hypotheses to evidence of effects, and ultimately to the quantification of impacts supported by sufficient evidence. This next part of research could be facilitated by ontology engineering and conceptual modeling, to ensure that gathered knowledge is reusable and the methodology adaptable across other exposure domains with potential indirect effects due to ecosystems disruptions (e.g., pesticide exposure). As such, the framework is also able to highlight gaps in the knowledge, and as such provides different types of insights as compared to traditional systematic reviews.

Given that our planetary system is a dynamic system and that in HIAs, health impacts are assessed as a function of frequency, severity, and probability (Lock 2000), adaptation and a spatiotemporal scale should also be integrated in the framework in the future. Impacts on human health due to decline in pollinators through nutrition could be unevenly distributed globally, with low‐income countries experiencing greater losses in food production, and medium‐ and high‐income countries facing more impacts on food consumption and mortality (Smith et al. 2022). Additionally, changes within a dynamic system triggers responses aiming to bring the system back in a state of equilibrium. These adaptations may affect the potential impacts and continue for years, which means that impacts depend on the evaluated timeframe. Finally, socioeconomic factors that may influence the population‐level vulnerability should be included, as economic development may allow the replacement of degraded natural systems with complex infrastructure that could minimize the impacts (Myers et al. 2013).

## Author Contributions

**Magdalini Stefanopoulou** contributed to methodology, formal analysis, investigation, visualization, validation, writing – original draft. **Tabea S. Sonnenschein** contributed to methodology, software development, formal analysis, writing – review and editing. **Florence Poulletier de Gannes** contributed to methodology and writing – review and editing. **Simon Scheider** was involved in software development and writing – review and editing. **Roel Vermeulen** provided supervision and writing – review and editing. **Martin Röösli** contributed to conceptualization, and writing – review and editing. **Anke Huss** was responsible for conceptualization, methodology, supervision, project administration, funding acquisition, validation and writing – review and editing. All authors have read and approved the final manuscript.

## Conflicts of Interest

The authors declare no conflicts of interest.

## Supporting information

Supplementary_material_revised.

## Data Availability

Only data extracted from the literature was used for this paper. All programming code is available at GitHub https://github.com/magdastef21/PHIA-automated-KG-RFEMF-fork.git.
