# Supplementary material for: Towards a Planetary Health Impact Assessment Framework: Exploring Expert Knowledge and Artificial Intelligence for a RF‐EMF Exposure Case‐Study
Source: Bioelectromagnetics. 2025 Dec 19;46(8):e70038. doi: 10.1002/bem.70038 (PMC12715659; doi:10.1002/bem.70038)
Supplement: Supplementary file 1 — Supplementary_material_revised. [file BEM-46-0-s001.docx]

**Towards a Planetary Health Impact Assessment Framework: Exploring Expert Knowledge and Artificial Intelligence for a RF-EMF Exposure Case-Study**

**Supplementary material**

Magdalini Stefanopoulou ^1*^, Tabea S. Sonnenschein ^1, 2, 3^, Florence Poulletier de Gannes ^4^, Simon Scheider ^2^, Roel Vermeulen ^1,3^, Martin Röösli ^5^, Anke Huss ^1^

^1^ Institute of Risk Assessment Sciences, Utrecht University, Utrecht, the Netherlands

^2^ Human Geography and Spatial Planning, Faculty of Geosciences, Utrecht University, Utrecht, the Netherlands

^3^ Julius Centre for Health Sciences and Primary Care, University Medical Centre Utrecht, Utrecht University, Utrecht, the Netherlands

^4^ Laboratoire de l’Intégration du Matériau au Système (IMS), Université Bordeaux, Talence, France

^5^ Department of Epidemiology and Public Health, Swiss Tropical and Public Health Institute, 4051 Basel, Switzerland

**Table of Contents**

[**Open AI’s Chat GPT**	2](#_Toc215487966)

[**Tables and Figures**	4](#_Toc215487967)

[**References**	29](#_Toc215487968)

[**Supplementary Table ST1.** List of questions as they were asked to the experts during the workshops.	4](#_Toc196484508)

**Supplementary Table** [**ST2**. The names, fields and countries of the experts that participated in our workshops and meetings	5](#_Toc196484509)

**Supplementary Table** [**S3.** Query components for search regarding the direct effects of RF-EMF exposure on living organisms.	6](#_Toc196484510)

**Supplementary Table** [**S4.** Query components for search regarding the indirect effects on human health through ecological consequences.	7](#_Toc196484511)

**Supplementary Table S5.** Overview of the ASReview screening studies and stopping criteria…...…....8 **Supplementary Table** [**S6.** Labels for the direct effects of RF-EMF exposure on living organisms……………..9](#_Toc196484512) **Supplementary Table** [**S7.** Labels for the indirect effects on human health through the ecological consequences.. …………………………………………………………………………….…………………..………………….10](#_Toc196484513) **Supplementary Table** **S8.** Parameters and performance metrics for the models used in this study………...…..11	**Supplementary Table** **S9.** Workflow summary for instances extraction…………………………………...…...11	**Supplementary Table** [**S10.** Instructions as they were given in the chatbot interface for the development of the ChatGPT KG……………………………………………………………………………………………………..12](#_Toc196484514)

**Supplementary Figure** [**SF1. The basic version of the KG as constructed based on the screened literature prior to the workshops with the experts.**	1](#_Toc203127459)3

**Supplementary Figure** [**SF2.** **Search strategy flow diagram for the direct effects of RF-EMF exposure on living organisms.**	1](#_Toc203127455)4

**Supplementary Figure** [**SF3.** **Search strategy flow diagram for the direct effects of RF-EMF exposure on living organisms.**	1](#_Toc203127456)5

**Supplementary Figure** [**SF4.** **Learning curve for the BERT model for direct effects.** The weighted F1-score for the best model is 0·64 in 5^th^ epoch. The F1-scores for the study types, study environment and moderators were the lowest among the individual labels.	1](#_Toc203127457)6

**Supplementary Figure** [**SF5.** **Learning curve for the BERT model for the indirect effects.** The weighted F1-score for the best model is 0·56 in 8^th^ epoch. The F1-score for the relationship was the lowest among the individual labels.	1](#_Toc203127458)6

**Supplementary Figure** [**SF6.** **Graph restricted to the extracted information related to study group of mammals.** The edges connecting the nodes represent the extracted hypothesized and reported associations. The edge width represents the number of articles stating the association with minimum 1 and maximum 27 articles.	1](#_Toc203127460)7

**Supplementary Figure** [**SF7.** **Graph restricted to the extracted information related to study group of animals.** The edges connecting the nodes represent the extracted hypothesized and reported associations. The edge width represents the number of articles stating the association with minimum 1 and maximum 7 articles.](#_Toc203127461)	18

**Supplementary Figure** [**SF8.** **Graph restricted to the extracted information related to study group of insects, including both pollinating and non-pollinating insects.** The edges connecting the nodes represent the extracted hypothesized and reported associations. The edge width represents the number of articles stating the association with minimum 1 and maximum 5 articles.](#_Toc203127462)	19

**Supplementary Figure** [**SF9**. **Graph restricted to the extracted information related to study group of plants.** The edges connecting the nodes represent the extracted hypothesized and reported associations. The edge width represents the number of articles stating the association with minimum 1 and maximum 7 articles.](#_Toc203127463)	20

**Supplementary Figure** [**SF10**. **Graph restricted to the extracted information related to study group of birds**. The edges connecting the nodes represent the extracted hypothesized and reported associations. The edge width represents the number of articles stating the association with minimum 1 and maximum 5 articles.](#_Toc203127464)	21

**Supplementary Figure** [**SF11. Graph restricted to the extracted information related to study group of aquatic organisms.** The edges connecting the nodes represent the extracted hypothesized and reported associations. The edge width represents the number of articles stating the association with minimum 1 and maximum 2 articles	2](#_Toc203127465)2

**Supplementary Figure** [**SF12.** **Graph restricted to the extracted information related to study group of nematodes.** The edges connecting the nodes represent the extracted hypothesized and reported associations. The edge width represents the number of articles stating the association with minimum 1 and maximum 2 articles.	2](#_Toc203127466)3

**Supplementary Figure** [**SF13**. **Graph restricted to the extracted information related to study group of bats.** The edges connecting the nodes represent the extracted hypothesized and reported associations. The edge width represents the number of articles stating the association with minimum 1 and maximum 3 articles.	2](#_Toc203127467)4

**Supplementary Figure** [**SF14.** **Graph restricted to the extracted information related to study group of amphibians and reptiles.** The edges connecting the nodes represent the extracted hypothesized and reported associations. The edge width represents the number of articles stating the association with minimum 1 and maximum 2 articles.	2](#_Toc203127468)5

**Supplementary Figure** [**SF15.** **Graph restricted to the extracted information related to study group of microorganisms**. The edges connecting the nodes represent the extracted hypothesized and reported associations. The edge width represents the number of articles stating the association with minimum 1 and maximum 4 articles.	2](#_Toc203127469)6

**Supplementary Figure** [**SF16.** **Graph restricted to the extracted information related to study group of invertebrate species.** The edges connecting the nodes represent the extracted hypothesized and reported associations. The extracted association were stated in 1 article.](#_Toc203127470)	27

**Supplementary Figure** [**SF17.** **Example knowledge graph constructed by ChatGPT using a subset of three open-access papers.**	2](#_Toc203127471)8

# **Open AI’s Chat GPT**

For our test case, OpenAI's ChatGPT (GPT-4-turbo version, accessed on November 2024) was tasked to extract and visualise the same information as Sonnenschein tool. We chose ChatGPT because we wanted to evaluate the feasibility of instruction-based extraction and visualisation in a way that did not require extensive programming or computational resources, making the approach more accessible to researchers from non-computational backgrounds. We provided ChatGPT with three open access studies [Cucurachi et al., 2013; Bodewein et al., 2022; Girela-Serrano et al., 2022] on the effects of RF-EMF on organisms and asked it to extract and visualize the relevant information, by providing instructions through the chatbot interface. The information requested for extraction and visualization corresponded to the labels used in Sonnenschein’ s tool [Sonnenschein et al., 2024]. S7 Table includes the instructions as they were given in the chatbot interface.

ChatGPT extracted and visualized the relevant information in a KG shown in Supplementary Figure SF17. The KG displayed only aggregated information, and it was impossible to check the individual initial extracted information. Nodes representing different elements of the KG (e.g. exposure, health effects, study group, etc.) were assigned distinct colors. Similar to the Sonnenschein tool-based KG, the node representing exposure was linked to the health effects and the health effects nodes were then linked to study groups, study types, study environments and/or moderators. Of note, when we attempted to reproduce the KG by providing the same instructions through the chatbot, we received KGs with various structures.

ChatGPT only provided access to aggregated information and did not require an expert to program it. However, it was not possible to confirm the reliability of the extracted and synthesized information. Specifically, our inability to access the underline algorithm combined with ChatGPT’s capability to ‘generate’ information make it impossible for us to confirm the reliability of the extracted and synthesized information. Another challenge of ChatGPT was the lack of reproducibility of its results. As its algorithms are changed and updated constantly, there is the risk that the generated results may vary over time, making it difficult for researchers to replicate or compare findings.

# **Tables and Figures**

**Supplementary Table ST1.** List of questions as they were asked to the experts during the workshops.

**Our experts were called to answer the following questions:**

a) After reviewing the hypotheses-based KG constructed from the umbrella review or formulated during the previous workshop, are there any additional nodes or edges that represent hypotheses about RF-EMF exposure and its direct effects on humans, animals, plants, or other organisms based on sound scientific reasoning?

b) Are there any additional nodes or edges representing hypotheses related to indirect effects on human health through ecological consequences, based on sound scientific reasoning?

c) Are there any nodes or edges in the existing graph that you strongly disagree with?

**Supplementary Table ST2.** The names, fields and countries of the experts that participated in our workshops and meetings.

| **Workshops** | **Experts** | **Field** | **Country** |
| --- | --- | --- | --- |
| 1^st^ Workshop | Maria Bouga | Agricultural Zoology, Entomology | Greece |
|  | Fani Hatjina | Biology, Apiculture | Greece |
|  | Menelaos Stavrinides | Agriculture, Environmental Sciences | Greece |
| 2^nd^ Workshop | Isabelle Lagroye | Life Sciences | France |
|  | Florence Poulletier de Gannes | Biological and Health Sciences | France |
|  | Christelle Lasbiez | Population and Ecosystems biology | France |
| 3^rd^ Workshop | Ken Karipidis | Physics, Epidemiology | Australia |
|  | Andrew Wood | Biophysics | Australia |
|  | Eric Van Rongen | Biology | Australia |
| 4^th^ Workshop | Arno Thielens | Engineering Physics | Belgium-New York |
| 5^th^ Workshop | Janine Schmidt | Biology | Germany |
| 6^th^ Workshop | Marek Czerwiński | Plant Ecology | Poland |

**Supplementary Table ST3.** Query components for search regarding the direct effects of RF-EMF exposure on living organisms.

| **Purpose** | **Query components** | **Results** |
| --- | --- | --- |
| RF-EMF and effects on human health | **Pub Med:** ((Radiofrequency electromagnetic fields) OR (rf emf)) AND (human) Filters: Review, Systematic Review, from 2000 - 2024 | 228 |
|  | **Web of Science:** ((((ALL=(radiofrequency electromagnetic fields)) OR ALL=(rf-emf)) AND ALL=(human)) AND DOP=(2000-01-01/2024-05-22)) AND DT=(Review) | 300 |
| RF-EMF and effects on insects | **Pub Med:** (((radiofrequency electromagnetic fields) OR (rf-emf)) OR (radiation)) AND (insects) Filters: Review, Systematic Review, from 2000 - 2024 | 3 |
|  | **Web of Science**: ((((ALL=(radiofrequency electromagnetic fields)) OR ALL=(radiation)) AND ALL=(insects)) AND DOP=(2000-01-01/2024-06-12)) AND DT=(Review) | 3 |
| RF-EMF and effects on animals | **Pub Med:** (((radiofrequency electromagnetic fields) OR (rf-emf)) ) AND (animals) Filters: Review, Systematic Review, from 2000 - 2024 | 79 |
|  | **Web of Science:** (ALL=(radiofrequency electromagnetic fields) OR ALL=(rf-emf) AND ALL=(animals)) AND DOP=(2000-01-01/2024-06-12)) AND (DT==("REVIEW")) | 57 |
| RF-EMF and effects on plants | **Pub Med:** (((radiofrequency electromagnetic fields) OR (rf-emf)) ) AND (plants) Filters: Review, Systematic Review, from 2000 - 2024 | 6 |
|  |  |  |
|  | **Web of Science:** (ALL=(radiofrequency electromagnetic fields) OR ALL=(rf-emf) AND ALL=(plants)) AND DOP=(2000-01-01/2024-06-12)) AND (DT==("REVIEW")) | 11 |

**Supplementary Table ST4.** Query components for search regarding the indirect effects on human health through ecological consequences.

| **Purpose** | **Query components** | **Results** |
| --- | --- | --- |
| Human health and pollinators | **Pub Med:** (human health) AND (pollinators population) OR (pollinators diversity) Filters: Review, Systematic Review, from 2000 - 2024 | 53 |
|  | **Web of Science:** human health (All Fields) and pollinators population (All Fields) or pollinators diversity (All Fields) and 2000-01-01/2024-08-26 (Publication Date) and Review (Document Type) | 65 |
| Human health and insects | **Pub Med:** (human health) AND (insects diversity) OR (insects population) Filters: Review, Systematic Review, from 2000 - 2024 | 1,229 |
|  | **Web of Science:** human health (All Fields) and insects diversity (All Fields) or insects population (All Fields) and 2000-01-01/2024-08-29 (Publication Date) and Review (Document Type) | 675 |
| Human health and birds | **Pub Med:** (human health) AND (birds population) OR (birds diversity) Filters: Review, Systematic Review, from 2000 - 2024 | 582 |
|  | **Web of Science:** human health (All Fields) and birds population (All Fields) or birds diversity (All Fields) and 2000-01-01/2024-08-26 (Publication Date) and Review (Document Type) | 471 |
| Human health and plants | **Pub Med:** (human health) AND (plants population) Filters: Review, Systematic Review, from 2000 - 2024 | 2,622 |
|  | **Web of Science: human health** (All Fields) and **plants population** (All Fields) and **2000-01-01/2024-08-26**(PublicationDate) and **Review** (Document Type) | 1,554 |

**Supplementary Table ST5.** Overview of the ASReview screening studies and stopping criteria.

| **Dataset Type** | **Total number of studies** | **Relevant Studies Retrieved** | **Last Relevant study** | **% of Dataset Screened When Stopped** | **Stopping Condition** |
| --- | --- | --- | --- | --- | --- |
| Direct RF-EMF Health Effects | 417 | 97 | 125 | 30.0% | ≥20–30% screened |
| Indirect effects -Insects | 1,538 | 5 | 11 | 7.2% (111 records) | 100 consecutively irrelevant |
| Indirect effects -Pollinators | 87 | 4 | 7 | 20.7% (18 records) | ≥20% screened |
| Indirect effects - Birds | 805 | 1 | 1 | 12.5% (101 records) | 100 consecutively irrelevant |
| Indirect effects - Plants | 3,321 | 3 | 12 | 3.3% (112 records) | 100 consecutively irrelevant |

**Supplementary Table ST6.** Labels for the direct effects of RF-EMF exposure on living organisms.

| **Labels** | **Description** |
| --- | --- |
| I-ExposureType | The type of exposure, specifically RF-EMF or any source of exposure to RF-EMF |
| I-HealthEffects | Health of behavior related outcomes observed on the studied organisms |
| I-AssocType | The reported or hypothesized association between the exposure and outcomes |
| I-Moderator | The variables that moderate the health effect of the exposure |
| I-StudyGroup | The types of organisms being studied |
| I-StudyType | The type of original study included (e.g., observational or experimental) |
| I-StudyEnvironment | The environment in which the original study was conducted (e.g., field or laboratory) |

**Supplementary Table ST7.** Labels for the indirect effects on human health through the ecological consequences.

| **Labels** | **Description** |
| --- | --- |
| I-DirectEffects | The direct effects on insects, birds, and plants as identified in the previous extraction. |
| I-EcoConsequence | Ecosystem services provided by insects, birds, and plants that could be disrupted due to the direct effects. |
| I-IndirectEffects | The indirect effects on human health resulting from the ecological consequences. |
| I-Relationship | The reported or hypothesized relationship between the direct and indirect effects. |
| I-StudyGroup | The types of organisms being studied |

**Supplementary Table ST8.** Parameters and performance metrics for the models used in this study. The table summarizes the training settings for BERT-based Named Entity Recognition (BERT-NER) and gradient boosting models, including validation strategies and models performance (F1-score).

| **Model Type** | **Parameters/Settings** | **Validation/Training Setup** | **Performance (F1-score)** |
| --- | --- | --- | --- |
| BERT-NER (Direct Effects) | Pretrained BERT cased base; token classification | 10% of labelled data for validation | Weighted F1-score = 0.64 |
| Epochs | 10 |  |  |
| Batch size | 35 |  |  |
| Learning rate | 0.000004 |  |  |
| BERT-NER (Indirect Effects) | Pretrained BERT cased base; token classification | 10% of labelled data for validation | Weighted F1-score = 0.56 |
| Epochs | 10 |  |  |
| Batch size | 35 |  |  |
| Learning rate | 0.000004 |  |  |
| Gradient Boosting (Direct Effects) | GradientBoostingClassifier (default parameters) | Repeated stratified cross-validation (10 splits, 3 repeats) | F1-score for predicting true instances = 0.76 |
|  |  |  | Weighted F1-score = 0.80 |
| Gradient Boosting (Indirect Effects) | GradientBoostingClassifier (default parameters) | Repeated stratified cross-validation (10 splits, 3 repeats) | F1-score for predicting true instances = 0.76 |
|  |  |  | Weighted F1-score = 0.80 |

**Supplementary Table ST9.** Workflow summary for instances extraction. The table shows the number of papers and sentences manually labelled, the instances predicted by the BERT-NER model, the subset of instances used to train the supervised classification models, and the final set of curated unique instances after gradient boosting classification.

| **Dataset of papers** | **Papers manually labelled** | **Sentences labelled** | **Predicted Instances (BERT-NER)** | **Instances used for supervised training** | **Final unique instances (Gradient Boosting)** |
| --- | --- | --- | --- | --- | --- |
| Direct Effects | 8 | 999 | 763,325 | 2,323 | 13,330 |
| Indirect Effects | 6 | 369 | 14,869 | 220 | 898 |

**Supplementary Table ST10.** Instructions as they were given in the chatbot interface for the development of the ChatGPT KG.

| **Type of Instruction** | **Instruction** |
| --- | --- |
| Objective of the project | “This project aims to extract and visualize information from the uploaded studies on RF-EMF exposure using a knowledge graph (KG). The extracted data will be categorized into distinct labels, and each will be visualized using a unique colour in the KG.” |
| Type of extracted information | “Extract and visualize information related to the following categories:   - Exposure Type: extract the type of RF-EMF exposure or any source of RF-EMF exposure in each study. - Health/Behavioural Outcomes: extract any health or behavioural effects observed in the organisms due to RF-EMF exposure. - Association Type: extract the type of association hypothesised or reported between RF-EMF exposure and the observed health/behavioural effects. - Study Group: extract the type of organisms or populations studied. - Study Type: extract the type of study design used (e.g., experimental, observational). - Study Environment: extract the environment in which the study was conducted. - Moderator: extract the variables that moderate the health effect of the exposure |
| Final output | “For the final output visualise the extracted information into a comprehensive knowledge graph.   - Use different colours for each of the six categories (Exposure Type, Health Effects, Association Type, Study Group, Study Type, Study Environment, Moderator) for clarity. - The KG should allow for easy exploration of the relationships between exposure types, health outcomes, moderators, study types, and environments. - Include a legend of the labels and corresponding colours - Include a general title.” |

**Supplementary Figure SF1. The basic version of the KG as constructed based on the screened literature prior to the workshops with the experts.**

**Supplementary Figure SF2.** **Search strategy flow diagram for the direct effects of RF-EMF exposure on living organisms.**

**Supplementary Figure SF3.** **Search strategy flow diagram for the direct effects of RF-EMF exposure on living organisms.**

**Supplementary Figure SF4.** **Learning curve for the BERT model for direct effects.** The x-axis represents the number of training iterations (epochs), while the y-axis shows the model’s performance across the epochs both in terms of the weighted total F1-score, as well as the F1-scores for each label separately. The weighted F1-score for the best model is 0·64 in 5^th^ epoch. The F1-scores for the study types, study environment and moderators were the lowest among the individual labels.

**Supplementary Figure SF5.** **Learning curve for the BERT model for the indirect effects.** The x-axis represents the number of training iterations (epochs), while the y-axis shows the model’s performance across the epochs both in terms of the weighted total F1-score, as well as the F1-scores for each label separately. The weighted F1-score for the best model is 0·56 in 8^th^ epoch. The F1-score for the relationship was the lowest among the individual labels.

**Supplementary Figure SF1.** **Graph restricted to the extracted information related to study group of mammals.** The edges connecting the nodes represent the extracted hypothesized and reported associations. The edge width represents the number of articles stating the association with minimum 1 and maximum 27 articles.

**Supplementary Figure SF2.** **Graph restricted to the extracted information related to study group of animals.** The edges connecting the nodes represent the extracted hypothesized and reported associations. The edge width represents the number of articles stating the association with minimum 1 and maximum 7 articles.

**Supplementary Figure SF3.** **Graph restricted to the extracted information related to study group of insects, including both pollinating and non-pollinating insects.** The edges connecting the nodes represent the extracted hypothesized and reported associations. The edge width represents the number of articles stating the association with minimum 1 and maximum 5 articles.

**Supplementary Figure SF4.** **Graph restricted to the extracted information related to study group of plants.** The edges connecting the nodes represent the extracted hypothesized and reported associations. The edge width represents the number of articles stating the association with minimum 1 and maximum 7 articles.

**Supplementary Figure SF5.** **Graph restricted to the extracted information related to study group of birds**. The edges connecting the nodes represent the extracted hypothesized and reported associations. The edge width represents the number of articles stating the association with minimum 1 and maximum 5 articles.

**Supplementary Figure SF6. Graph restricted to the extracted information related to study group of aquatic organisms.** The edges connecting the nodes represent the extracted hypothesized and reported associations. The edge width represents the number of articles stating the association with minimum 1 and maximum 2 articles

**Supplementary Figure SF7. Graph restricted to the extracted information related to study group of nematodes.** The edges connecting the nodes represent the extracted hypothesized and reported associations. The edge width represents the number of articles stating the association with minimum 1 and maximum 2 articles.

**Supplementary Figure SF8.** **Graph restricted to the extracted information related to study group of bats.** The edges connecting the nodes represent the extracted hypothesized and reported associations. The edge width represents the number of articles stating the association with minimum 1 and maximum 3 articles.

**Supplementary Figure SF9.** **Graph restricted to the extracted information related to study group of amphibians and reptiles.** The edges connecting the nodes represent the extracted hypothesized and reported associations. The edge width represents the number of articles stating the association with minimum 1 and maximum 2 articles.

**Supplementary Figure SF10.** **Graph restricted to the extracted information related to study group of microorganisms**. The edges connecting the nodes represent the extracted hypothesized and reported associations. The edge width represents the number of articles stating the association with minimum 1 and maximum 4 articles.

**Supplementary Figure SF11.** **Graph restricted to the extracted information related to study group of invertebrate species.** The edges connecting the nodes represent the extracted hypothesized and reported associations. The extracted association were stated in 1 article.

**Supplementary Figure SF12.** **Example knowledge graph constructed by ChatGPT using a subset of three open-access papers.**

# **References**

1. **Bodewein L**, Dechent D, Graefrath D, Kraus T, Krause T, Driessen S. 2022. Systematic review of the physiological and health-related effects of radiofrequency electromagnetic field exposure from wireless communication devices on children and adolescents in experimental and epidemiological human studies. PLoS One [Internet] 17(6):e0268641. Available from: <http://dx.doi.org/10.1371/journal.pone.0268641>
2. **Cucurachi S,** Tamis WLM, Vijver MG, Peijnenburg WJGM, Bolte JFB, de Snoo GR. 2013. A review of the ecological effects of radiofrequency electromagnetic fields (RF-EMF). Environ Int [Internet] 51:116–140. Available from: <http://dx.doi.org/10.1016/j.envint.2012.10.009>
3. **Girela-Serrano BM,** Spiers ADV, Li R, Gangadia S, Toledano MB, Di Simplicio M. 2022. Impact of mobile phones and wireless devices use on children and adolescents’ mental health: a systematic review. Eur Child Adolesc Psychiatry [Internet]. Available from: <https://doi.org/10.1007/s00787-022-02012-8>
